# Supplementary material for: Complexome profiling of the Chlamydomonas psb28 mutant reveals TEF5 as an early PSII assembly factor
Source: Plant Cell. 2025 Mar 17;37(6):koaf055. doi: 10.1093/plcell/koaf055 (PMC12164590; doi:10.1093/plcell/koaf055)
Supplement: koaf055_Supplementary_Data [file koaf055_supplementary_data.zip › psb28_interactive.zip!/plots/intensity/qualityPlots/peptides/_Cre06.g263300.t1.2.html]

### Cre06.g263300.t1.2

Trivial name:   
SeqCoverage [median]: 29.50  
Mass: 25.54 kDa  
  
 

| Sequence | MissedCleavage | Mass [Da] | wtIntensity | muIntensity | Score |
| --- | --- | --- | --- | --- | --- |
| EGESEEDYAR | 0 | 1183.46 | 11680.37 | 10215.31 | 138.98 |
| IQASVTAAR | 0 | 915.51 | 112550.23 | 132302.36 | 159.58 |
| KEGESEEDYAR | 1 | 1311.56 | 19567.01 | 19983.58 | 150.06 |
| KIQASVTAAR | 1 | 1043.61 | 875.65 | 0.00 | 57.86 |
| QPMLLEAVNK | 0 | 1141.62 | 2962.48 | 17585.09 | 145.16 |
| QPMLLEAVNKVK | 1 | 1368.78 | 3833.81 | 1176.67 | 87.67 |
| SPTYAEHLSVIK | 0 | 1343.71 | 11825.75 | 24478.41 | 193.49 |
| SVVRKEGESEEDYAR | 2 | 1752.83 | 3959.79 | 2445.16 | 82.10 |
| VMRPLELMTPLLITPGFTGK | 1 | 2213.23 | 7121.76 | 35576.25 | 116.39 |
|  |  |  | 174376.8 | 243762.8 |  |
